# Intermittently Scanned Continuous Glucose Monitoring Data of Polish Patients from Real-Life Conditions: More Scanning and Better Glycemic Control Compared to Worldwide Data

**DOI:** 10.1089/dia.2021.0034

**Published:** 2021-08-04

**Authors:** Jerzy Hohendorff, Janusz Gumprecht, Malgorzata Mysliwiec, Dorota Zozulinska-Ziolkiewicz, Maciej Tadeusz Malecki

**Affiliations:** ^1^ Department of Metabolic Diseases, Jagiellonian University Medical College, Krakow, Poland.; ^2^ Department of Internal Medicine, Diabetology and Nephrology, Medical University of Silesia, Katowice, Poland.; ^3^ Department of Pediatrics, Diabetology and Endocrinology, Medical University of Gdansk, Poland.; ^4^ Department of Internal Medicine and Diabetology, Poznan University of Medical Science, Poland.

**Keywords:** Self-monitoring of blood glucose, Continuous glucose monitoring systems, Intermittently scanned CGM, Real-world data

## Abstract

***Background:*** Randomized trials and observational studies have shown that the use of FreeStyle Libre^®^ intermittently scanned continuous glucose monitoring system (isCGMS) is associated with improved glycemic indices and quality of life.

***Materials and Methods:*** In this retrospective, real-world data analysis, we described country-specific glucometrics among isCGMS users from Poland and compared them with international data. The analyzed time period for the Polish data ranged between August 2016 and August 2020, and the analyzed time period for the international data ranged from September 2014 to August 2020.

***Results:*** Data from the Polish population were collected from 10,679 readers and 92,627 sensors with 113 million automatically recorded glucose readings. The worldwide database included information from 981,876 readers and 11,179,229 sensors with 13.1 billion glucose readings. On average, the users of isCGMS from Poland performed substantially more scans/day (21.2 ± 14.2 vs. 13.2 ± 10.7), achieved lower eHbA1c (7.0% ± 1.2% vs. 7.5% ± 1.5%), and spent more time-in-range (TIR) (64.2% ± 17.3% vs. 58.1% ± 20.3%) and less time-above-range (TAR) (29.7% ± 18.0% vs. 36.6% ± 21.3%) (*P* < 0.0001 for all comparisons). Moreover, they were more likely to achieve TIR >70% (36.3% vs. 28.8%), but spent more time-below-range (TBR) (4.7% vs. 3.6%). Our results confirmed that analyzed glucometrics improve as the scan rate frequency increases. However, at a similar scanning frequency to the comparative group, users from Poland achieved lower eHbA1c, higher TIR, and lower TAR, but higher TBR.

***Conclusions:*** We report more scanning and better glycemic control in isCGMS users in Poland than worldwide. The cause of this observation remains unknown. Our data also show that in real-life practice, a large number of patients may be willing to perform scanning more frequently than it is usually assumed.

## Introduction

It was estimated in 2019 that, there were 463 million people with diabetes worldwide.^1^ In Poland, the number of people living with diabetes is almost 3 million, including >200,000 patients diagnosed with type 1 diabetes mellitus (T1DM).^1,2^ Interestingly, Poland has experienced one of the fastest growths in incidence rates of TIDM worldwide over the last three decades.^3,4^ While the reason for this phenomenon remains uncertain, it seems to be associated with the socioeconomic changes occurring after 1989, a year of major political reform in Poland.

It is well proven that the self-monitoring of blood glucose (SMBG) with a glucose meter increases the efficiency of intensive insulin therapy in T1DM. For example, performing more frequent glucose measurements was associated with improved glycemic control as measured by the HbA1c level.^5,6^ Local and international guidelines suggest at least four measurements per day in patients on multiple daily insulin injections or treated with insulin pumps. However, a more frequent target of between 6 and 10 measurements per day seems to be an optimal SMBG frequency.^7,8^

Recent analysis of the pharmacological records reported an SMBG test rate of 3.99 tests/day and 2.61 tests/day, respectively, for Polish patients with T1DM and type 2 diabetes mellitus (T2DM) treated with insulin.^9^ Similar data can be observed in other European countries.^10^ Some of the likely reasons for measuring blood glucose levels less frequently than recommended are due to the invasiveness and the pain associated with finger pricks as well as the inconvenience and social embarrassment associated with testing.^11^

An alternative for SMBG performed by glucose meters is continuous glucose monitoring systems (CGMS) that can be either intermittently scanned (isCGMS or intermittently viewed, ivCGMS) or real time (rtCGMS).^12^ In Poland, the only commercially available isCGMS is the FreeStyle Libre^®^ flash glucose monitoring system (Abbott Diabetes Care, Inc.). Randomized clinical trials and observational studies have shown that the use of FreeStyle Libre is safe and associated with improved glycemic indices with respect to parameters such as time spent in range (TIR), time-below-range (TBR) or time-above-range (TAR), and the quality of life.^13–16^ The FreeStyle Libre system was introduced to the European market in 2014.

In Poland, this system has been available since August 2016. The most important factor limiting the disseminated use of the FreeStyle Libre system within the Polish population has been the cost of this device. Up until late 2019, these devices were not covered under national health insurance policies and patients had to pay for the device “out-of-pocket.” After November 2019, a limited reimbursement was introduced for T1DM patients younger than 18 years.^17^

In Poland, there are no formal recommendations for specific CGMS use. In general, patients make a decision on a specific device based on the predicted cost and characteristics of the product (accuracy, calibration need, compatibility with insulin pump, etc.)—FreeStyle Libre or rtCGMS. Enlite and Dexcom sensors are reimbursed in patients <26 years of age diagnosed with T1DM treated with insulin pump and diagnosed with hypoglycemia unawareness. The Eversense system is not reimbursed in Poland, at all.^17^ Of note, test strips for glucose meters and insulin analogs are almost fully reimbursed in Poland for patients with T1DM regardless of their age.^18^

In this retrospective, real-world data (RWD) analysis, our aim was to describe country-specific glucometrics among Freestyle Libre users from Poland and compare these results with international data.

## Materials and Methods

### Sensors and readers

The FreeStyle Libre system is an isCGMS with a sensor that measures interstitial glucose levels for up to 2 weeks.^19^ The FreeStyle Libre Reader is used to quickly scan the sensor. A reader collects and displays glucose data, including the current glucose level, glucose trend arrow, and the last 8-h history of glucose levels. Data collected by the FreeStyle Libre Reader are stored for up to 90 days and can be uploaded by patients using the complementary software to generate their personal reports (LibreView^®^; Abbott Diabetes Care, Inc.). Once the patient's consent is obtained, such data are altered so personal information is removed and other data are collected safely and anonymously stored in a cloud database. Thus, the dataset used in this study was built from the anonymous data that were uploaded from users of the reader and the desktop software. Abbott Diabetes Care has provided the author's access to anonymously processed data supporting findings from both the Polish and worldwide cohorts. Neither demographic nor personal data, including age, sex, type of diabetes, diabetes duration, type of hypoglycemic therapy, education, and socioeconomic status of the FreeStyle Libre users, were available to the authors.

### Scanning details

Scanning frequency was assessed for each sensor by dividing the sum of scans by the duration of sensor use.

### Glycemic measures

Glucose range for assessment of TIR, TBR, and TAR was defined as 70–180 mg/dL (3.9–10.0 mmol/L), <70 mg/dL (<3.9 mmol/L), and >180 mg/dL (>10.0 mmol/L), respectively, in accordance with the international consensus.^20^ We also evaluated time spent in very high glucose and very low ranges defined as >250 mg/dL (13.9 mmol/L) and <54 mg/dL (<3.0 mmol/L). An estimation of A1c was performed based on the ADAG study formula (eHbA1c).^21^

### Comparison of Polish and global data

The glycemic control indices were compared between Polish and international data. The analyzed time period for the Polish data ranged between August 2016 and August 2020, and the analyzed time period for the international data ranged from September 2014 to August 2020. In addition, the Polish and global datasets were compared with recently published data from Spain, a country with a similar population size and territory as Poland.^22^

### Statistical analysis

The cumulative frequency of scan rates was calculated for each 10% of available readers and basic descriptive statistics were calculated. The glycemic control indices were then analyzed as a function of the 10 scan-frequency groups of readers. Differences across groups were assessed by ANOVA or Kruskall–Wallis test. To compare Polish and worldwide data, *t* testing and *U* testing were performed. Differences between Polish and global data were also analyzed between five groups with equivalent scan rates, across the range of scan frequencies 10–30 measurements/day. Given the large sample size and multiple analyses, a *P* < 0.001 was considered to be significant.

## Results

### Data collection

For the analysis, Polish data were collected from 10,679 readers and 92,627 sensors with 22.3 million glucose scans and 113 million automatically recorded glucose readings. The worldwide database included information from 981,876 readers and 11,179,229 sensors with 1.75 billion scans and 13.1 billion automatically recorded glucose readings. Most readers were provided by European countries (59.9%); among them, Germany (19.6%), France (14.2%), and Italy (3.8%) were the most frequent with the readers. Readers provided by North American countries accounted for 17.6%.

### Frequency of glucose testing

**FIG. 1.**
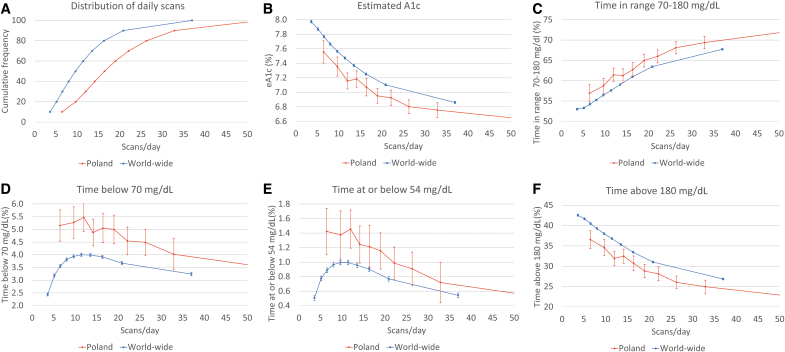
**(A)**. Cumulative distribution of daily scans by readers. Association between daily scan frequency and **(B)** mean estimated A1c, **(C)** mean time in range, **(D)** median time below 70 mg/dL, **(E)** median time at or below 54 mg/dL, and **(F)** mean time above 180 mg/dL. Each point represents 1068 or 10% of readers for a total of 22.3 million scans and 28.2 million monitoring hours. Polish data—red line; global data—blue line. For Polish data, each point is 1068 or 10% (*n* = 10,679). For global data, each point is 98,188 or 10% (*n* = 981,876). Color images are available online.

FreeStyle Libre users in Poland performed an average of 21 scans per day (median 18, [interquartile range 12–26], Fig. 1A). Glucose control measures, including estimated A1c, TIR, TBR, and TAR by scan rate groups, are shown in Table 1.

**Table 1.** Glucose Control Measures in the Polish Population Presented According to the Scan Rate Group

Scan rate (scans/day)	Days of monitoring (*n*)	Estimated A1c		<54 mg/dL		<70 mg/dL		70–180 mg/dL		>180 mg/dL		>250 mg/dL		Glucose SD	Glucose CV
		(%)	(mmol/mol)	(%)	(min/day)	(%)	(min/day)	(%)	(h/day)	(%)	(h/day)	(%)	(h/day)	(mg/dL)	(%)
6.5 ± 1.4	97.0 ± 115.9	7.6 ± 1.5	59	1.4	20.5	5.2	74.2	56.9	13.7	36.6	8.8	16.5	4.0	70.4	40.2
9.7 ± 0.7	119.5 ± 145.0	7.4 ± 1.3	57	1.4	19.8	5.3	75.9	58.7	14.1	34.6	8.3	14.1	3.4	67.9	40.5
12.0 ± 0.6	134.5 ± 153.6	7.2 ± 1.1	55	1.5	21.0	5.5	78.8	61.5	14.8	31.9	7.7	11.8	2.8	65.1	40.3
14.1 ± 0.6	136.8 ± 145.8	7.2 ± 1.1	55	1.2	17.9	4.9	70.2	61.3	14.7	32.5	7.8	11.7	2.8	64.3	39.9
16.4 ± 0.7	135.6 ± 143.2	7.1 ± 1.2	54	1.2	17.5	5.0	72.7	62.7	15.1	30.7	7.4	10.8	2.6	62.0	39.4
19.0 ± 0.8	128.1 ± 144.7	7.0 ± 1.0	53	1.2	16.6	5.0	72.0	65.0	15.6	28.8	6.9	9.3	2.2	60.0	38.8
22.1 ± 1.0	115.9 ± 127.7	6.9 ± 1.0	52	1.0	14.2	4.5	65.5	66.0	15.8	28.1	6.8	8.9	2.1	58.3	37.9
26.3 ± 1.5	102.6 ± 114.0	6.8 ± 0.9	51	1.0	13.1	4.5	64.7	68.1	16.3	26.0	6.2	7.6	1.8	56.4	37.6
32.9 ± 2.5	80.1 ± 86.3	6.8 ± 1.0	51	0.7	10.4	4.0	57.9	69.4	16.7	25.0	6.0	7.0	1.7	54.3	36.6
53.2 ± 17.3	49.4 ± 63.6	6.6 ± 1.0	49	0.5	7.8	3.5	50.9	72.3	17.3	22.5	5.4	6.0	1.4	50.7	35.0

Each scan group consists of *n* = 1068, except the highest group that contains *n* = 1067. All data are shown as mean, but <54 and <70 mg/dL (median).

CV, coefficient of variation; SD, standard deviation.

### Estimated A1c

Mean estimated A1c was 7.0% ± 1.2%, which is the target for this parameter in Poland and most other countries.^7,8^ Estimated A1c was significantly lower in patients with the highest scan frequency in comparison to the lowest scanning group—6.6% ± 1.0% versus 7.6% ± 1.5% (*P* < 0.0001; Fig. 1B).

### Time in range

Mean TIR was 64.2% ± 17.3% (15 h 24 min/day). A mean TIR higher than 70%, the current therapeutic goal for most patients with diabetes,^20^ was observed in the highest scanning group. In this group of users, TIR was 27.1% higher compared to the lowest scanning group—72.3% ± 15.4% versus 56.9% ± 21.1% (*P* < 0.0001; Fig. 1C).

### Time in hypoglycemia

Median time spent in hypoglycemia, defined as glucose level <70 mg/dL, was 67.7 min/day (4.7%). The observed median TBR was different between the lowest and the highest scan rate group, 3.5% (51 min/day) and 5.2% (1 h 14 min/day), respectively, (*P* < 0.0001; Fig. 1D). Similar observations were made for hypoglycemia, <54 mg/dL—1.4% ± 3.1% (21 min/day) and 0.5% ± 2.4% (8 min/day), for the lowest and highest scan rate group, respectively (*P* < 0.0001; Fig. 1E). Interestingly, in the Polish data, the longest time spent in hypoglycemia, both below 54 and 70 mg/dL, characterized the group ranked third from the bottom in terms of scanning rate (1.5% corresponding to 21.0 min/day and 5.5% corresponding to 78.8 min/day, respectively).

### Time in hyperglycemia

The mean TAR (>180 mg/dL) was 29.7% ± 18.0% (7 h 7 min/day). Comparing the lowest and highest scan rate users, TAR ranged from 36.6% ± 21.5% (8 h 48 min/day) to 22.5% ± 16.5% (5 h 24 min/day), (*P* < 0.0001; Fig. 1F). Mean time spent in hyperglycemia higher than 250 mg/dL was 10.4% ± 11.0% (2 h 29 min/day).

### Glycemic variability

Glycemic variability expressed as standard deviation (SD) or coefficient of variation (CV) was significantly lower in patients performing more frequent scans. The highest and lowest scan rate groups mean SD reached 50.7 and 70.4 mg/dL, respectively, while for CV, it varied between 35.0% and 40.2%, respectively (*P* < 0.0001 for both comparisons).

### Comparison of Polish and worldwide data

Polish and worldwide data are presented in Figure 1. Glucometrics of the two cohorts are shown in Table 2. On average, users of isCGM from Poland performed substantially more scans per day (21.2 vs. 13.2), were characterized by lower eHbA1c (7.0% vs. 7.5%), and spent more TIR (64.2% vs. 58.1%) and less TAR (29.7% vs. 36.6%) (*P* < 0.0001 for all comparisons). Moreover, users from Poland were more likely to achieve TIR >70% (36.3% vs. 28.8%). However, they spent more time in hypoglycemic range. The median time below 54 mg/dL and time below 70 mg/dL were higher in users from Poland than worldwide users: 1.06% versus 0.82% and 4.70% versus 3.59%, respectively (*P* < 0.0001 for both comparisons).

**Table 2.** Mean Glucometrics of Polish and Worldwide Users

	Poland	Worldwide	*P*
No. of readers (*n*)	10 679	981 876	N/A
No. of sensors (*n*)	92 627	11 179 229	N/A
Days of monitoring (*n*)	110.0 ± 129.9	138.5 ± 163.7	<0.0001
Scan rate (*n*/days)	21.2 ± 14.2	13.2 ± 10.7	<0.0001
eHbA1c (%)	7.04 ± 1.16	7.49 ± 1.46	<0.0001
TB54 (%)	1.06	0.82	<0.0001
TB70 (%)	4.70	3.59	<0.0001
TIR (%)	64.2 ± 17.3	58.1 ± 20.3	<0.0001
TA180 (%)	29.7 ± 18.0	36.6 ± 21.3	<0.0001
TA250 (%)	10.4 ± 11.0	14.5 ± 15.0	<0.0001
SD (mg/dL)	60.9 ± 20.8	64.0 ± 23.2	<0.0001
CV (%)	38.6 ± 8.7	37.5 ± 9.0	<0.0001
% of patients with TIR >70%	36.3	28.8	<0.0001

Data are shown as mean ± SD, but TB54, TB70 (median).

TIR, time-in-range.

**FIG. 2.**
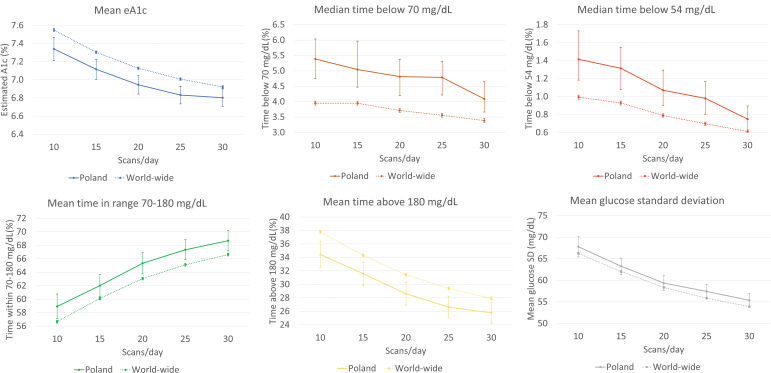
Polish (solid line) and worldwide (dashed line) glucometrics trends versus daily scan frequency. Poland bin size: 1067, worldwide bin size: 98,187. Error bars show the 99.9% confidence interval of the mean. Since medians are presented for TB70 and TB54, their confidence intervals are 99.9% confidence interval for median. Color images are available online.

We also compared the Polish and international data across the range of scan frequencies (10–30 measurements/day) using five scan intervals. The results are summarized in Table 3 and Figure 2. In addition, in Table 3, we have cited the Spanish data published in 2020.^22^ Among the provided data sets, all glucometrics, including the TIR, TAR, TBR, and eHbA1c, improved as scan rate frequency increased.

**Table 3.** Polish, Spanish,^a^ and Worldwide Scan Frequencies and Glucometrics

Scan rate (scans/day)	Devices (*n*)			eHbA1c (%)			<54 mg/dL (min/day)			<70 mg/dL (min/day)			70–180 mg/dL (h/day)			>180 mg/dL (h/day)			>250 mg/dL (h/day)			Glycemic variability SD (mg/dL)			Glycemic variability CV (%)		
	PL	WW	SP	PL	WW	SP	PL	WW	SP	PL	WW	SP	PL	WW	SP	PL	WW	SP	PL	WW	SP	PL	WW	SP	PL	WW	SP
10	1067	98187	2296	7.3	7.5	7.5	20.4	14.3	N/A	77.5	56.9	N/A	14.1	13.6	13.2	8.3	9.1	9.1	3.3	3.6	N/A	67.8	66.0	69.7	40	39	42
15	1067	98187	2296	7.1	7.3	7.3	18.9	13.4	N/A	72.6	56.8	N/A	14.9	14.4	13.9	7.6	8.2	8.5	2.7	3.0	N/A	63.3	61.8	65.4	40	38	40
20	1067	98187	2296	6.9	7.1	7.2	15.4	11.4	N/A	69.3	53.5	N/A	15.7	15.1	14.4	6.9	7.5	8.1	2.2	2.6	N/A	59.4	58.1	63.1	39	37	39
25	1067	98187	2296	6.8	7.0	7.2	14.1	10.1	N/A	69.0	51.3	N/A	16.2	15.6	14.8	6.4	7.1	7.8	1.9	2.3	N/A	57.4	55.6	60.9	38	36	38
30	1067	98187	2296	6.8	6.9	7.1	10.8	8.8	N/A	58.9	48.8	N/A	16.5	16.0	15.1	6.2	6.7	7.6	1.8	2.2	N/A	55.4	53.7	59.2	37	35	38

All glucometrics shown as mean, but <54 and <70 mg/dL (median). Since Spanish data for TBR were published as mean, but not median, here, we do not cite these values.^22^ Data highlighted in gray are statistically significantly different (*P* < 0.001, comparison Polish and worldwide data).

^a^ Spanish data published in Gomez-Peralta et al.^22^

TBR, time-below-range.

Interestingly, users from Poland achieved a lower HbA1c at the same scan rate frequency when compared to users from other countries (Table 3 and Fig. 2). Furthermore, this difference corresponds to more time spent in the target glycemic range and less time spent in hyperglycemia by patients from Poland. However, FreeStyle Libre users from Poland, when compared to worldwide users, spent more time in the hypoglycemic range and glycemic variability expressed as CV, but not SD, is higher (*P* < 0.001 for most comparisons among scan rate frequencies—highlighted in gray in Table 3). Of note, a higher scan rate, lower eHbA1c, and more TIR were associated with less TBR in cohorts from Poland and worldwide. Users from Poland when compared to those from Spain (no formal statistics available) seem to achieve lower eHbA1c, more TIR, less TAR, and lower glycemic variability.

## Discussion

In this retrospective study, we report Polish-specific glucose measures in isCGMS users in real-life settings. We confirmed findings from earlier reports and described the association between scanning frequency and glycemic control indices in the Polish cohort.^15,22–24^ In all reported populations, a high scan rate is associated with lower eHbA1c and higher TIR, as well as less time spent in hypoglycemia.^15,22–24^ However, new and unexpected findings are related to differences found between the analyzed cohorts. First, such differences were found in terms of scanning frequency as users from Poland performed many more daily glucose checks. In addition, crude mean eHbA1c is lower in the Polish population than eHbA1c seen in Spanish, Belgian, and worldwide isCGMS users.

Moreover, at the same scanning frequencies, patients from Poland achieved ∼a 0.2% lower HbA1c than users from both the international cohort and from Spain. This finding is supported by a longer TIR and shorter TAR, as well. This may be of clinical significance as a recently published prospective study performed in patients with T2DM provided evidence of a strong inverse relationship between TIR and all-cause and cardiovascular mortality.^25^ However, to date, there has not been a similar study on long-term outcomes in T1DM.

The Polish data also showed the phenomenon visible earlier on other countries' data regarding the recorded hypoglycemic time, as the highest TBR levels were found in patients performing 6–12 measurements/day. In the users with less frequent scanning, the recorded TBR was shorter, which could probably be explained by the fact that they have higher glucose levels and their TAR was much longer, and as a result, they experienced less hypoglycemia.

The patients from Poland experienced more hypoglycemia compared to users from other countries. Moreover, they experienced higher glycemic variability expressed as CV. The observed higher glycemic variability, an independent risk factor for hypoglycemia, and more time spent below range were apparent across all the scan frequency rates. This could be the result of aggressive countermeasures taken by Polish users to prevent or treat hyperglycemia as well as differences in the distribution of diabetes types and models of hypoglycemic therapy.^26^

The finding of a lower eHbA1c in isCGMS users in Poland is in line with the PolPeDiab study on glycemic control in almost 1000 Polish children with T1DM treated in three university centers. The mean HbA1c level in the PolPeDiab study was 7.6% and it was up to 1.2% lower than the HbA1c reported in similar studies, including high-quality registries. This could suggest the presence of country-specific factors influencing HbA1c.^27–31^ These factors might include different models of outpatient care, the training of health care professionals, patient education, lifestyle factors, the impact of socioeconomic status, and reimbursement schemes.

However, the potential causes of the observed lower eHbA1c in Polish isCGMS user groups with the same scanning frequencies are difficult to define as no demographic data were available. For example, there are no data on age, type of diabetes, diabetes duration, age, or details of hypoglycemic therapy. Furthermore, there are no data on any previous training performed on the optimal use of FreeStyle Libre and no data on the number of visits in outpatient clinics or telehealth visits.

In November 2019 when the FreeStyle Libre started to be reimbursed in Poland, for the vast majority of the study period, this technology was unavailable for most individuals with diabetes in Poland. The authors' observations from clinical practice are that the system was used more frequently by patients with a higher educational level and socioeconomic status. Nevertheless, unless there is a similar study with unblinded demographic data, this remains just a hypothesis as country-specific heterogeneity could not be explored.

Interestingly, the observed results from a real-world setting differ from clinical trial data, especially in terms of TBR. In the IMPACT study, T1DM patients with the HbA1c level comparable to this study spent on average 2.0 h/day in the hypoglycemic range, which is much more in comparison to recent Polish, Spanish, and international data.^13,22^ Of note, in the IMPACT study, the observed scanning frequency of 15.1 scans/day is less than shown by the Polish (21.2), and similar to Spanish (13.2) and international population data (13.2).^13,22^ Nevertheless, it should be emphasized that due to the different nature of the study design, any comparison between RWD observations and results of randomized trials should be done with great caution.

There is a well-proven association of the number of daily glucose scans and glycemic indices. Recommendations on the frequency of measurements should be individualized to patients specifically to achieve set glycemic targets. The average daily scan rate among Polish patients was 21; this is substantially higher than target recommendations proposed by the Endocrine Society.^32^ Over the period of this retrospective analysis, national guidelines in Poland recommended at least six daily measurements, preferably between 8 and 12, for both glucometer measurements and, beginning in 2018, isCGM scans in pediatric cases of T1DM. For the adult T1DM patients, at least four glucometer measurements were recommended daily without any specific reference to isCGM scanning.

In this study, we did not analyze data regarding scan rate with respect to the time of day and longitudinal data on scanning frequency. However, previous studies have shown that the number of daily scans is the highest in the first few days of FreeStyle Libre use and then stabilized and many more measurements are performed during the daytime than at nights.^13,15,33^

This study provides data that could lead to the future discussion on the suggested number of daily scans, which would bring clinical benefits, especially for patients treated with insulin. Based on Polish data, it seems that the optimal frequency of daily scanning is within the range of 15–20. At this scanning frequency rate, the mean achieved eHbA1c was close to or lower than 7.0% (the current eHbA1c goal) and the mean TIR was >65%, very near the current recommended target. Mean TIR >70% was achieved by a group of patients performing, on average, 53.2 scans per day, an unreasonably high number for most.

There are some limitations related to this study. The retrospective, observational nature of the analysis may be prone to typical biases related with this type of study design. Moreover, the study was limited by its masked nature and unavailability of demographic data. Most likely, the provided data, even with the huge number of readers, do not represent the majority of patients with diabetes requiring insulin therapy in Poland. With 10,000 readers, this would equate to no >1.0% of all patients with diabetes treated with insulin. Finally, the analyzed period partially covered a few months of the start of COVID-19 pandemic, and with varying lockdown strategies adopted by countries around the globe, we cannot exclude that some observations were biased by unforeseen changes in patients' everyday life or quality of life, as well as access to health care.

## Conclusion

Data obtained from isCGMS users in Poland showed that sensors were scanned more frequently, achieving lower eHbA1c as well as higher TIR, lower TAR, and higher TBR in comparison to worldwide users. This difference remained when comparing data with the same scanning frequency. Due to the nature of this study, possible causes of the observed differences are not apparent. Our data also show that in real-life practice, a large number of patients may be willing to perform scanning more frequently than it is usually assumed.

## Data Availability

The data that support the findings of this study are available from Abbott Diabetes Care and restrictions apply to the availability of these data, which are not publicly available. Data are, however, available from the authors upon reasonable request and with permission from Abbott Diabetes Care.

## Bioethics Committee Approval

The data analyzed in the study were depersonalized and anonymously stored. The data were collected for the purpose of research according to standard research rules and EULA terms. No bioethical committee approval was required for the study.
